# Supplementary material for: The Learning Transfer of Dementia Training Program Participants: Its Antecedents and Mediating Effect on the Job Competency of Geriatric Caregivers
Source: Healthcare (Basel). 2023 Nov 19;11(22):2991. doi: 10.3390/healthcare11222991 (PMC10671796; doi:10.3390/healthcare11222991)
Supplement: Supplementary file 1 [file healthcare-11-02991-s001.zip › healthcare-2662883-supplementary.pdf]

healthcare MDPI

Supplementary

Learning Transfer of the Dementia Training Program Participants: Its Antecedents and Mediating Effect on Job Competency of Geriatric Caregivers

Supplementary S1: Dementia Specialist Training Curriculum

(1) Basic Course - 40 Hours

The basic course in dementia specialist training consists of 40 hours and covers topics such as symptoms of dementia, treatment of dementia, fundamentals of dementia care, dementia care techniques, family caregiving for dementia, observation, and record-keeping.

	Course	Contents
	**Orientation**	Understanding Dementia Specialist Training
**Understanding Dementia**	Symptoms of Dementia	Concept and Types of Dementia
		Cognitive Symptoms of Dementia
		Behavioral Symptoms of Dementia
		Physical Symptoms of Dementia
	Treatment of Dementia	Cognitive Function Improvement
		Treatment of Behavioral Symptoms
		Non-Pharmacological Treatment
**Understanding Caregiving**	Fundamentals of Dementia Care	Goals and Principles of Caregiving
		Caregiver Ethics
	Dementia Care Techniques	Communication and Relationship Building
		Daily Life Care
		Utilizing Welfare Devices
		Care for Behavioral Symptoms
		Care for Physical Symptoms
		Nutrition Management
		Emergency Care
	Dementia Family Care	Understanding Dementia Families
		Support Services for Dementia Families
	Observation and Recording	Recording for Dementia Patients and Families

(2) Facility Course - 20 Hours

The facility course is intended for caregivers in residential care facilities, including those working in day and night care settings. It focuses on providing care for elderly individuals with dementia within a facility setting.

	Course	Contents
**Facility Care**	Introduction to Dementia Care in Facilities	Goals and Principles of Dementia Care in Facilities
		Practical Aspects of Relationship Building: Case Studies
	Dementia Care Techniques in Facilities	Facility Environment and Safety Management
		Implementation of Activities to Enhance Daily Life
		Implementation of Cognitive Stimulation Activities
		Implementation of Physical Stimulation Activities

	Care for End-Stage Dementia Patients
	Practical Aspects of Dementia Patient Records in Facilities

Supplementary S2: Mediating Effect of Learning Transfer (Near, Far) on Job Competency

<**Table S1**> Results of the Mediating Effect of Near Learning Transfer on Communication Competency (n=279)

	Model	Level 1		Level 2		Level 3	
		**B**	**t**	**B**	**t**	**B**	**t**
	(Constant)		2.394		2.756		2.011
Control variable	Sex (Male_dum)	-.117	-1.948	.005	.081	.050	.822
	Age	.008	.143	.028	.488	.025	.466
	Training level	.043	.761	.087	1.434	.070	1.241
	Work experience	-.024	-.448	-.056	-.973	-.047	-.869
	Training experience	.000	-.002	.075	1.335	.075	1.427
	Monthly income	.072	1.028	-.017	-.227	-.045	-.633
	Training point	.013	.259	-.004	-.075	-.009	-.178
	Work type (Facility_dum)	.098	1.322	-.074	-.939	-.112	-1.500
Antecedent variable	Learning motivation	-.038	-.648	-.022	-.345	-.007	-.122
	Self-efficacy	.131	2.032*	.179	2.598*	.129	1.986*
	Training content	.019	.283	.020	.271	.012	.182
	Training method	.101	1.382	-.044	-.563	-.083	-1.122
	Role of instructor	.101	1.296	.284	3.415**	.246	3.144**
	Manager support	-.107	-1.431	-.053	-.659	-.012	-.159
	Peer support	.076	1.174	.111	1.594	.082	1.252
	Transfer opportunity	.300	4.775***	.043	.641	-.071	-1.085
	Transfer intention	.275	4.152***	.317	4.478***	.213	3.103**
Mediating variable	Near transfer					.382	6.144***
Goodness of fit		F=10.586***, R2=.408 (Adjusted, .370)		F= 7.229***, R2=.320 (Adjusted,.276),		F= 9.885***, R2=.406(Adjusted,.365), ΔR2=.086	

* p<.05, ** p<.01, *** p<.001

<**Table S2**> Results of the Mediating Effect of Near Learning Transfer on Problem-solving Competency (n=279)

Model		Level 1		Level 2		Level 3	
		**B**	**t**	**B**	**t**	**B**	**t**
	(Constant)		2.394		3.319		2.608
Control variable	Sex (Male_dum)	-.117	-1.948	-.067	-1.023	-.022	-.351
	Age	.008	.143	-.029	-.500	-.032	-.588
	Training level	.043	.761	.095	1.554	.078	1.369
	Work experience	-.024	-.448	-.104	-1.799	-.095	-1.750
	Training experience	.000	-.002	.025	.449	.025	.480
	Monthly income	.072	1.028	.106	1.395	.078	1.097
	Training point	.013	.259	-.027	-.493	-.032	-.624
	Work type (Facility_dum)	.098	1.322	.066	.826	.029	.380
Antecedent variable	Learning motivation	-.038	-.648	-.140	-2.177*	-.125	-2.077
	Self-efficacy	.131	2.032*	.248	3.557***	.198	3.004**
	Training content	.019	.283	-.024	-.335	-.032	-.465
	Training method	.101	1.382	-.095	-1.206	-.134	-1.804
	Role of instructor	.101	1.296	.190	2.261*	.151	1.917
	Manager support	-.107	-1.431	.102	1.257	.143	1.877
	Peer support	.076	1.174	.020	.285	-.009	-.141
	Transfer opportunity	.300	4.775***	.167	2.454*	.052	.777
	Transfer intention	.275	4.152***	.223	3.117**	.117	1.698
Mediating variable	Near transfer					.384	6.125***
Goodness of fit		F=10.586***, R2=.408 (Adjusted, .370)		F= 6.819***, R2=.308 (Adjusted,.262),		F= 9.426***, R2=.395(Adjusted,.353), ΔR2=.087	

* p<.05, ** p<.01, *** p<.001

<**Table S3**> Results of the Mediating Effect of Near Learning Transfer on Self-development Competency (n=279)

Model		Level 1		Level 2		Level 3	
		**B**	**t**	**B**	**t**	**B**	**t**
	(Constant)		2.394		.666		-.053
Control variable	Sex (Male_dum)	-.117	-1.948	-.213	-3.437**	-.177	-2.964**
	Age	.008	.143	.071	1.308	.069	1.324
	Training level	.043	.761	.130	2.242*	.117	2.104*
	Work experience	-.024	-.448	-.050	-.904	-.042	-.805
	Training experience	.000	-.002	.101	1.887	.101	1.975*
	Monthly income	.072	1.028	.200	2.768**	.178	2.568*
	Training point	.013	.259	.014	.272	.010	.203
	Work type (Facility_dum)	.098	1.322	.246	3.239**	.216	2.965**
Antecedent variable	Learning motivation	-.038	-.648	.156	2.561*	.168	2.880**
	Self-efficacy	.131	2.032*	.142	2.140*	.101	1.590
	Training content	.019	.283	-.046	-.668	-.052	-.787
	Training method	.101	1.382	-.187	-2.493*	-.219	-3.030**
	Role of instructor	.101	1.296	.051	.637	.020	.260
	Manager support	-.107	-1.431	.008	.098	.041	.548
	Peer support	.076	1.174	.271	4.063***	.248	3.872***
	Transfer opportunity	.300	4.775***	.116	1.795	.024	.366
	Transfer intention	.275	4.152***	.275	4.042***	.190	2.835**
Mediating variable	Near transfer					.308	5.063***
Goodness of fit		F=10.586***, R^2=.408 (Adjusted, .370)		F= 9.175***, R^2=.374 (Adjusted,..333)		F= 10.907***, R^2=.430(Adjusted,.391), ΔR^2=.056	

* p<.05, ** p<.01, *** p<.001

<**Table S4**> Results of the Mediating Effect of Near Learning Transfer on Interpersonal Relationship Competency (n=279)

Model		Level 1		Level 2		Level 3	
		**B**	**t**	**B**	**t**	**B**	**t**
	(Constant)		2.394		1.119		.489
Control variable	Sex (Male_dum)	-.117	-1.948	-.251	-4.094***	-.219	-3.674***
	Age	.008	.143	-.019	-.352	-.021	-.404
	Training level	.043	.761	.136	2.377*	.125	2.249*
	Work experience	-.024	-.448	-.072	-1.320	-.066	-1.242
	Training experience	.000	-.002	.082	1.539	.082	1.595
	Monthly income	.072	1.028	.124	1.728	.104	1.502
	Training point	.013	.259	-.002	-.034	-.005	-.107
	Work type (Facility_dum)	.098	1.322	.261	3.465**	.234	3.212**
Antecedent variable	Learning motivation	-.038	-.648	.080	1.321	.090	1.547
	Self-efficacy	.131	2.032*	.132	2.011*	.096	1.507
	Training content	.019	.283	.051	.743	.046	.691
	Training method	.101	1.382	-.023	-.308	-.050	-.700
	Role of instructor	.101	1.296	-.005	-.063	-.032	-.424
	Manager support	-.107	-1.431	.047	.620	.076	1.036
	Peer support	.076	1.174	.215	3.256**	.194	3.039**
	Transfer opportunity	.300	4.775***	.044	.685	-.038	-.593
	Transfer intention	.275	4.152***	.295	4.382***	.220	3.279**
Mediating variable	Near transfer					.273	4.491***
Goodness of fit		F=10.586***, R^2=.408 (Adjusted, .370)		F= 9.709***, R^2=.387 (Adjusted,.347)		F= 10.963***, R^2=.431(Adjusted,.392), ΔR^2=.044	

* p<.05, ** p<.01, *** p<.001

<**Table S5**> Results of the Mediating Effect of Near Learning Transfer on Skill Competency (n=279)

Model		Level 1		Level 2		Level 3	
		**B**	**t**	**B**	**t**	**B**	**t**
	(Constant)		2.394		2.726		2.085
Control variable	Sex (Male_dum)	-.117	-1.948	-.109	-1.667	-.071	-1.132
	Age	.008	.143	.039	.680	.037	.666
	Training level	.043	.761	.093	1.509	.079	1.340
	Work experience	-.024	-.448	-.069	-1.184	-.061	-1.098
	Training experience	.000	-.002	-.049	-.863	-.049	-.901
	Monthly income	.072	1.028	.077	1.003	.053	.728
	Training point	.013	.259	.027	.485	.022	.426
	Work type (Facility_dum)	.098	1.322	.078	.966	.046	.599
Antecedent variable	Learning motivation	-.038	-.648	.042	.645	.054	.874
	Self-efficacy	.131	2.032*	.167	2.381*	.125	1.845
	Training content	.019	.283	-.066	-.895	-.072	-1.023
	Training method	.101	1.382	.112	1.403	.079	1.035
	Role of instructor	.101	1.296	.081	.961	.049	.601
	Manager support	-.107	-1.431	.143	1.754	.177	2.266*
	Peer support	.076	1.174	-.053	-.747	-.077	-1.140
	Transfer opportunity	.300	4.775***	.035	.507	-.062	-.908
	Transfer intention	.275	4.152***	.265	3.684***	.177	2.484*
Mediating variable	Near transfer					.322	4.996***
Goodness of fit		F=10.586***, R^2=.408 (Adjusted, .370)		F= 6.563***, R^2=.299 (Adjusted,.254)		F= 8.154***, R^2=.361(Adjusted,.317), ΔR^2=.061	

* p<.05, ** p<.01, *** p<.001

<**Table S6**> Results of the Mediating Effect of Near Learning Transfer on Work Ethics Competency (n=279)

Model		Level 1		Level 2		Level 3	
		**B**	**t**	**B**	**t**	**B**	**t**
	(Constant)		2.394		6.824		6.331
Control variable	Sex (Male_dum)	-.117	-1.948	-.031	-.459	.005	.081
	Age	.008	.143	.078	1.318	.076	1.328
	Training level	.043	.761	.067	1.070	.054	.892
	Work experience	-.024	-.448	.107	1.785	.114	1.981
	Training experience	.000	-.002	-.151	-2.596*	-.151	-2.695**
	Monthly income	.072	1.028	.125	1.594	.103	1.358
	Training point	.013	.259	-.073	-1.300	-.077	-1.424
	Work type (Facility_dum)	.098	1.322	.022	.262	-.009	-.107
Antecedent variable	Learning motivation	-.038	-.648	.164	2.483*	.176	2.761**
	Self-efficacy	.131	2.032*	-.093	-1.297	-.134	-1.914
	Training content	.019	.283	-.067	-.894	-.073	-1.010
	Training method	.101	1.382	.024	.288	-.008	-.096
	Role of instructor	.101	1.296	-.099	-1.143	-.130	-1.553
	Manager support	-.107	-1.431	.057	.685	.090	1.117
	Peer support	.076	1.174	.147	2.027*	.124	1.764
	Transfer opportunity	.300	4.775***	.135	1.922	.042	.602
	Transfer intention	.275	4.152***	.249	3.363**	.164	2.230*
Mediating variable	Near transfer					.308	4.630***
Goodness of fit		F=10.586***, R^2=.408 (Adjusted, .370)		F= 5.437***, R^2=.262 (Adjusted,.213),		F=6.728***, R^2=.318(Adjusted,.271) ΔR^2=.056	

* p<.05, ** p<.01, *** p<.001

<**Table S7**> Results of the Mediating Effect of Far Learning Transfer on Communication Competency (n=279)

Model		Level 1		Level 2		Level 3	
		**B**	**t**	**B**	**t**	**B**	**t**
	(Constant)		1.000		2.756		2.562
Control variable	Sex (Male_dum)	-.093	-1.556	.005	.081	.037	.597
	Age	.006	.120	.028	.488	.026	.473
	Training level	.040	.707	.087	1.434	.073	1.273
	Work experience	-.129	-2.413*	-.056	-.973	-.012	-.223
	Training experience	.092	1.763	.075	1.335	.044	.816
	Monthly income	.140	2.001*	-.017	-.227	-.065	-.894
	Training point	-.094	-1.863	-.004	-.075	.028	.534
	Work type (Facility_dum)	.119	1.620	-.074	-.939	-.115	-1.515
Antecedent variable	Learning motivation	.023	.387	-.022	-.345	-.030	-.490
	Self-efficacy	.125	1.938	.179	2.598*	.137	2.076*
	Training content	-.069	-1.027	.020	.271	.043	.624
	Training method	.152	2.080*	-.044	-.563	-.095	-1.268
	Role of instructor	.168	2.172*	.284	3.415**	.227	2.847**
	Manager support	-.086	-1.150	-.053	-.659	-.024	-.312
	Peer support	.121	1.863	.111	1.594	.070	1.053
	Transfer opportunity	.122	1.945	.043	.641	.002	.030
	Transfer intention	.370	5.609***	.317	4.478***	.192	2.696**
Mediating variable	Far transfer					.338	5.340***
Goodness of fit		F=10.728***, R^2=.411 (Adjusted, .373)		F= 7.229***, R^2=.320 (Adjusted,.276),		F=9.131***, R^2=.387(Adjusted,.345) ΔR^2=.067	

* p<.05, ** p<.01, *** p<.001

<**Table S8**> Results of the Mediating Effect of Far Learning Transfer on Problem-solving Competency (n=279)

	Model	Level 1		Level 2		Level 3	
		**B**	**t**	**B**	**t**	**B**	**t**
	(Constant)		1.000		3.319		3.201
Control variable	Sex (Male_dum)	-.093	-1.556	-.067	-1.023	-.053	-.814
	Age	.006	.120	-.029	-.500	-.030	-.520
	Training level	.040	.707	.095	1.554	.089	1.468
	Work experience	-.129	-2.413*	-.104	-1.799	-.085	-1.467
	Training experience	.092	1.763	.025	.449	.012	.211
	Monthly income	.140	2.001*	.106	1.395	.085	1.123
	Training point	-.094	-1.863	-.027	-.493	-.013	-.241
	Work type (Facility_dum)	.119	1.620	.066	.826	.049	.608
Antecedent variable	Learning motivation	.023	.387	-.140	-2.177*	-.143	-2.245*
	Self-efficacy	.125	1.938	.248	3.557***	.230	3.295**
	Training content	-.069	-1.027	-.024	-.335	-.014	-.197
	Training method	.152	2.080*	-.095	-1.206	-.118	-1.486
	Role of instructor	.168	2.172*	.190	2.261*	.165	1.964
	Manager support	-.086	-1.150	.102	1.257	.114	1.420
	Peer support	.121	1.863	.020	.285	.002	.033
	Transfer opportunity	.122	1.945	.167	2.454*	.149	2.191*
	Transfer intention	.370	5.609***	.223	3.117**	.169	2.243*
Mediating variable	Far transfer					.147	2.204*
Goodness of fit		F=10.728***, R^2=.411 (Adjusted, .373)		F= 6.819***, R^2=.308 (Adjusted,.262),		F=6.805***, R^2=.320(Adjusted,.273) ΔR^2=.013	

* p<.05, ** p<.01, *** p<.001

<**Table S9**> Results of the Mediating Effect of Far Learning Transfer on Self-development Competency (n=279)

	Model	Level 1		Level 2		Level 3	
		**B**	**t**	**B**	**t**	**B**	**t**
	(Constant)		1.000		.666		.468
Control variable	Sex (Male_dum)	-.093	-1.556	-.213	-3.437**	-.193	-3.163**
	Age	.006	.120	.071	1.308	.070	1.309
	Training level	.040	.707	.130	2.242*	.122	2.136*
	Work experience	-.129	-2.413*	-.050	-.904	-.022	-.408
	Training experience	.092	1.763	.101	1.887	.082	1.544
	Monthly income	.140	2.001*	.200	2.768**	.170	2.383**
	Training point	-.094	-1.863	.014	.272	.034	.666
	Work type (Facility_dum)	.119	1.620	.246	3.239**	.221	2.948**
Antecedent variable	Learning motivation	.023	.387	.156	2.561*	.151	2.530*
	Self-efficacy	.125	1.938	.142	2.140*	.115	1.762
	Training content	-.069	-1.027	-.046	-.668	-.032	-.463
	Training method	.152	2.080*	-.187	-2.493*	-.220	-2.958**
	Role of instructor	.168	2.172*	.051	.637	.015	.190
	Manager support	-.086	-1.150	.008	.098	.026	.341
	Peer support	.121	1.863	.271	4.063***	.246	3.727***
	Transfer opportunity	.122	1.945	.116	1.795	.090	1.411
	Transfer intention	.370	5.609***	.275	4.042***	.196	2.777**
Mediating variable	Far transfer					.213	3.409**
Goodness of fit		F=10.728***, R^2=.411 (Adjusted, .373)		F= 9.175***, R^2=.374 (Adjusted,.333),		F=9.663***, R^2=.401(Adjusted,.359) ΔR^2=.027	

* p<.05, ** p<.01, *** p<.001

<**Table S10**> Results of the Mediating Effect of Far Learning Transfer on Interpersonal Relationship Competency (n=279)

Model		Level 1		Level 2		Level 3	
		**B**	**t**	**B**	**t**	**B**	**t**
	(Constant)		1.000		1.119		.955
Control variable	Sex (Male_dum)	-.093	-1.556	-.251	-4.094***	-.234	-3.856***
	Age	.006	.120	-.019	-.352	-.020	-.378
	Training level	.040	.707	.136	2.377*	.129	2.282*
	Work experience	-.129	-2.413*	-.072	-1.320	-.049	-.899
	Training experience	.092	1.763	.082	1.539	.065	1.239
	Monthly income	.140	2.001*	.124	1.728	.099	1.386
	Training point	-.094	-1.863	-.002	-.034	.015	.296
	Work type (Facility_dum)	.119	1.620	.261	3.465**	.239	3.209**
Antecedent variable	Learning motivation	.023	.387	.080	1.321	.076	1.270
	Self-efficacy	.125	1.938	.132	2.011*	.109	1.682
	Training content	-.069	-1.027	.051	.743	.063	.934
	Training method	.152	2.080*	-.023	-.308	-.050	-.676
	Role of instructor	.168	2.172*	-.005	-.063	-.035	-.447
	Manager support	-.086	-1.150	.047	.620	.063	.831
	Peer support	.121	1.863	.215	3.256**	.194	2.950**
	Transfer opportunity	.122	1.945	.044	.685	.022	.346
	Transfer intention	.370	5.609***	.295	4.382***	.229	3.254**
Mediating variable	Far transfer					.179	2.874**
Goodness of fit		F=10.728***, R^2=.411 (Adjusted, .373)		F= 9.709***, R^2=.387 (Adjusted,.347),		F=9.883***, R^2=.406(Adjusted,.365) ΔR^2=.019	

* p<.05, ** p<.01, *** p<.001

<**Table S11**> Results of the Mediating Effect of Far Learning Transfer on Skill Competency (n=279)

Model		Level 1		Level 2		Level 3	
		**B**	**t**	**B**	**t**	**B**	**t**
	(Constant)		1.000		2.726		2.543
Control variable	Sex (Male_dum)	-.093	-1.556	-.109	-1.667	-.082	-1.296
	Age	.006	.120	.039	.680	.037	.670
	Training level	.040	.707	.093	1.509	.081	1.368
	Work experience	-.129	-2.413*	-.069	-1.184	-.032	-.564
	Training experience	.092	1.763	-.049	-.863	-.075	-1.363
	Monthly income	.140	2.001*	.077	1.003	.037	.490
	Training point	-.094	-1.863	.027	.485	.053	1.001
	Work type (Facility_dum)	.119	1.620	.078	.966	.044	.557
Antecedent variable	Learning motivation	.023	.387	.042	.645	.035	.562
	Self-efficacy	.125	1.938	.167	2.381*	.131	1.923
	Training content	-.069	-1.027	-.066	-.895	-.046	-.646
	Training method	.152	2.080*	.112	1.403	.068	.880
	Role of instructor	.168	2.172*	.081	.961	.033	.400
	Manager support	-.086	-1.150	.143	1.754	.167	2.121*
	Peer support	.121	1.863	-.053	-.747	-.087	-1.270
	Transfer opportunity	.122	1.945	.035	.507	.000	-.004
	Transfer intention	.370	5.609***	.265	3.684***	.159	2.161*
Mediating variable	Far transfer					.286	4.385***
Goodness of fit		F=10.728***, R^2=.411 (Adjusted, .373)		F= 6.563***, R^2=.299 (Adjusted,.254),		F=7.700***, R^2=.348(Adjusted,.303) ΔR^2=.048	

* p<.05, ** p<.01, *** p<.001

<**Table S12**> Results of the Mediating Effect of Far Learning Transfer on Work Ethics Competency (n=279)

Model		Level 1		Level 2		Level 3	
		**B**	**t**	**B**	**t**	**B**	**t**
	(Constant)		1.000		6.824		6.724
Control variable	Sex (Male_dum)	-.093	-1.556	-.031	-.459	-.015	-.229
	Age	.006	.120	.078	1.318	.077	1.313
	Training level	.040	.707	.067	1.070	.061	.973
	Work experience	-.129	-2.413	.107	1.785	.128	2.138*
	Training experience	.092	1.763	-.151	-2.596*	-.167	-2.866**
	Monthly income	.140	2.001	.125	1.594	.102	1.300
	Training point	-.094	-1.863	-.073	-1.300	-.058	-1.027
	Work type (Facility_dum)	.119	1.620	.022	.262	.002	.022
Antecedent variable	Learning motivation	.023	.387	.164	2.483*	.161	2.447*
	Self-efficacy	.125	1.938	-.093	-1.297	-.114	-1.587
	Training content	-.069	-1.027	-.067	-.894	-.056	-.747
	Training method	.152	2.080*	.024	.288	-.002	-.020
	Role of instructor	.168	2.172*	-.099	-1.143	-.127	-1.465
	Manager support	-.086	-1.150	.057	.685	.072	.861
	Peer support	.121	1.863	.147	2.027*	.127	1.756
	Transfer opportunity	.122	1.945	.135	1.922	.115	1.637
	Transfer intention	.370	5.609***	.249	3.363**	.187	2.415*
Mediating variable	Far transfer					.166	2.414*
Goodness of fit		F=10.728***, R^2=.411 (Adjusted, .373)		F= 5.437***, R^2=.262 (Adjusted,.213),		F=5.554***, R^2=.278(Adjusted,.223) ΔR^2=.016	

* p<.05, ** p<.01, *** p<.001
